# The Impact of a New Interleukin-2-Based Immunotherapy Candidate on Urothelial Cells to Support Use for Intravesical Drug Delivery

**DOI:** 10.3390/life10100231

**Published:** 2020-10-05

**Authors:** Lisa Schmitz, Belinda Berdien, Edith Huland, Petra Dase, Karin Beutel, Margit Fisch, Oliver Engel

**Affiliations:** 1 Department of Urology, University Medical Center Hamburg-Eppendorf (UKE), 20251 Hamburg, Germany; p.dase@uke.de (P.D.); k.beutel@uke.de (K.B.); j.matney@uke.de (M.F.); o.engel@uke.de (O.E.); 2 Immunservice GmbH, 20251 Hamburg, Germany; berdien@immunservice.com (B.B.); huland@immunservice.com (E.H.)

**Keywords:** interleukin-2, urothelial cells, intravesical, immunotherapy, bladder cancer

## Abstract

(1) Background: The intravesical instillation of interleukin-2 (IL-2) has been shown to be very well tolerated and promising in patients with bladder malignancies. This study aims to confirm the use of a new IL-2 containing immunotherapy candidate as safe for intravesical application. IL-2, produced in mammalian cells, is glycosylated, because of its unique solubility and stability optimized for intravesical use. (2) Materials and Methods: Urothelial cells and fibroblasts were generated out of porcine bladder and cultured until they reached second passage. Afterwards, they were cultivated in renal epithelial medium (REM) and Dulbecco’s modified Eagles medium (DMEM) with the IL-2 candidate (IMS-Research) and three more types of human interleukin-2 immunotherapy products (IMS-Pure, Natural IL-2, Aldesleukin) in four different concentrations (100, 250, 500, 1000 IU/mL). Cell proliferation was analyzed by water soluble tetrazolium (WST) proliferation assay after 0, 3, and 6 days for single cell culture and co-culture. (3) Results: Proliferation assays showed that all IL-2 products induced very similar cultivation results and none of the IL-2 variants had a negative impact on the proliferation of urothelial cells and fibroblast in either concentration. (4) Conclusion: Human recombinant glycosylated IL-2 as well as human non-glycosylated IL-2 have no negative influence on the tissue cell proliferation of porcine urothelial cells and fibroblasts in vitro and represent a promising and innovative potential intravesical therapy candidate for patients in high need.

## 1. Introduction

### 1.1. Interleukin-2 and Its Diverse Potential

Interleukin-2 (IL-2), first discovered in 1976 as the T cell growth factor [1], has today been completely characterized and analyzed in several experimental and clinical studies [2]. IL-2 plays a crucial role in the immune system by functioning as an autocrine and paracrine mediator in the expansion and differentiation of lymphocytes as well as natural killer cells and other cells of the cytotoxic cell system [3]. Furthermore, various other cytokines and factors are influenced by the effects of IL-2, such as the tumor-necrosis-factor alpha (TNFa) or interferons (IFN) [4,5]. It was found that its signaling pathways are much more complex than previously thought. Already in the late 1990s, Nelson and Willerford claimed the IL-2 receptor pathway to impact “the survival, effector function, and apoptosis” of immune cells depending on apparent conditions [5,6]. This characteristic is mainly caused by its ability to promote different intracellular pathways, like mitogen-activated protein kinase (MAPK) or phosphatidylinositol-3-kinase (Pi3K) [7]. An important role regarding the diversity of pathways lies in its receptor (IL-2R), which is divided into three subunits, of which only the alpha chain (CD25) is specific. Both the beta (CD122) and gamma (CD132) subunits present an affinity for other cytokines like interleukin-15 as well [6]. The heterotrimeric structure of the IL-2R is associated with diverse states of affinity according to the parts of the receptor presented [8]. Consequently, various signaling pathways linked to the IL-2R produce diverging results as the TH1 or TH2 lymphocyte differentiation depending on the present cytokine microenvironment [7]. It is also decisive for the promotion of either CD4+ T regulatory cells or CD8+ cytotoxic T cells, which is important because CD4+ cells were found to limit the anti-tumor effects of IL-2, while CD8+ cells promote these outcomes [9,10].

### 1.2. IL-2 Influencing Tissue Cells

The diverse potential of IL-2 influencing physiological as well as pathological processes has transpired over the years and led to a shift of focus in current scientific interests [11]. Primarily known as the T cell growth factor, the impact of IL-2 on tissue cells and its potential therapeutical effects has become a new center of attention. It was found that, not only hematopoietic cells, but also many other cell lines were able to express at least parts of the heterotrimeric IL-2R [12,13,14,15,16,17,18]. Furthermore, Mishra and colleagues were able to show that the concentration of IL-2 is as relevant for the fate of tissue cells. They described different effects of IL-2 on intestinal mucosa depending on the concentration used. In low doses of less than 50 IU/mL, proliferation was induced, but with double the concentration, the effect turned out to be counterproductive and apoptosis was induced [19].

### 1.3. IL-2 in Cancer Therapy

On the basis of this knowledge, many studies found diverse types of cancer to be sensitive towards a cytokine therapy and confirmed the positive impact of IL-2 with regard to immuno-therapeutical cancer therapy [20]. Thus, IL-2 was the first approved immunological treatment for cancer [21,22]. Over the past years, the narrow therapeutic window has exhibited an extensive limitation [23]. As high bolus therapy is needed to activate the high affinity tri-complex and IL-2 presents a very short and bi-phasic in vivo half-time, many toxic adverse effects in high dose therapy, especially at systemic application, occur [24,25,26]. A well-balanced treatment with close meshed observation is needed to ensure minimal complications and maximal regression. Consequently, the IL-2 therapy is still reserved for a few forms of cancer like metastatic renal cell carcinoma [27,28,29,30] and intralesional application to treat metastatic melanoma [31,32,33,34]. To address toxicity, local applications are very well tolerated and may be promising to treat locally accessible tumors such as melanoma or bladder cancer. Nevertheless, new investigations also focus on alternatives to overcome this hurdle by developing for example IL-2 types with different binding characteristics. They aim to enlarge the spectrum of target cells for systemically applicated IL-2 with a tolerable outcome for patients [35].

### 1.4. Bladder Cancer

Bladder cancer is one of the most common cancer types worldwide, appearing as non-muscle-invasive bladder cancer (NMIBC) in over 75% of cases [36,37], and is associated with lifelong risks of recurrence [38,39]. It was found to be highly immunogenic owing to somatic mutations [40]. Although studies proved IL-2 to also be effective against NMIBC [41,42,43,44,45,46], owing to severe side effects and the necessary expertise of highly specialized centers, this cytokine-based therapy has limited usability [33]. Besides the transurethral removement, most commonly, the adjuvant Bacillus Calmette-Guerin (BCG) intravesical installation immunotherapy has become, and still is [47,48,49], the gold standard for high risk NMIBC worldwide [50,51].

Our study aims to contribute to the completion of the understanding of the IL-2 impact in therapeutic approaches, especially bladder cancer. Thus, our results intend to support the implementation of this high potential candidate as a safe alternative in bladder cancer therapy.

### 1.5. Rational to Develop an Innovative Interleukin-2 Candidate for Intravesical Use

For intravesical use, there is a need to develop an innovative interleukin-2 candidate in mammalian cells, which results in a cytokine, very similar to the natural interleukin-2. The intravesical application of therapeutic agents into the bladder requires solubility, stability, and optimal compatibility of the therapeutic agent within the bladder environment and particularly within urine solutions. Natural interleukin-2 has been shown in vivo to be biologically active in the bladder environment [46]. Natural interleukin-2, for example, interleukin-2 produced by the German Red Cross from the purified supernatant of stimulated human lymphocytes, is not available any more. Those products are preferred for several reasons. The recombinant marketed product, Aldesleukin, has no glycosylation as it is produced in *E. coli*, and thus differs significantly in stability and solubility from the natural interleukin-2.

Aldesleukin is not identical to interleukin-2, but is an analogue of interleukin-2 (the chemical name is desalanyl-1, serine-125 human interleukin-2) and is incompatible to dilution in sodium solutions; this is very likely to interfere with the intravesical application and bioactivity in the bladder. The Summary of Product Characteristics (SmPC) of Proleukin/Aldesleukin recommends avoiding the use of sodium chloride 0.9% for dilution, because this may result in the incomplete delivery of bioactivity and/or formation of biologically inactive protein and because it may cause increased aggregation. The therapeutic results of Aldesleukin may not reflect the real therapeutic potential of interleukin-2 because of the loss of bioactivity in the bladder environment [52].

## 2. Materials and Methods

### 2.1. Isolation and Cultivation of Cells

The porcine bladder tissue used was stored in a freezing medium at −80 °C and carefully thawed at 37 °C for 10 min prior to each test. After rinsing the tissue with a 10% phosphate buffered saline solution (PBS) including 1% penicillin/streptomycin (P/S), it was fragmented into 2 × 2 mm pieces. Each part was laid into a six-well-plate. The pieces were dried for 10 min and afterwards covered with a medium. To promote the growth of urothelial cells, a premixed renal epithelial medium (REM) supplemented with 1% P/S was used. In order to generate fibroblast growth, Dulbecco’s modified Eagles medium (DMEM) supplemented with 10% fetal calf serum (FCS) and 1% P/S was added to the bladder tissue. Prepared plates were incubated in a humidified incubator at 37 °C and 5% carbon dioxide (CO_2_).

Cultures were fed every second to third day by removing the old medium, rinsing every well with some added PBS solutions and subsequently adding a new medium depending on the promoted cell type. After three weeks, cell layers reached a confluence of about 70% and were harvested. Similarly, cultivated cells of further passages were fed every two to three days and harvested at a confluence of about 70%.

The harvesting was performed by first removing the old medium and rinsing it with a PBS solution. Thereafter, 1 mL Accutase cell detachment solution was added and incubated for ten minutes. The enzymatical reaction was stopped by adding about 5 mL PBS 10% FCS. By centrifugating all of the generated solution at 1100 rpm for ten minutes and discarding the supernatant, a cell pellet was gained. Cells were resuspended with 1 mL of proper medium and 5 × 10^3^ to 1 × 10^4^ cells suspended in 20 mL medium were distributed in cultivation flasks. Every further harvesting out of cultivation flasks was performed similarly to this procedure, except that 4 mL detachment solution instead of 1 mL was used. All experiments were performed in technical and biological triplicates.

The cultivation of cytotoxic T-lymphocytes (CTLL) was performed with an RPMI (Roswell park memorial institute) medium, augmented with 1% Pyruvat and 500 international units (IU) per well IMS-Research. These non-attachable cells were fed by centrifuging the whole cultivation solution for 10 min at 1100 rpm, discarding the supernatant and resuspending the cell pellet with a fresh IL-2 infused medium.

### 2.2. Used Interleukin-2 Types

For our study, we used four different types of interleukin-2. The potential immunotherapy candidate IMS-Research, which was compared with other IL-2 types, is partially glyosylated and produced in the Chinese hamster ovary (CHO). A highly purified IL-2 version of CHO cells was used for the testing as well (IMS-Pure). Another tested IL-2 type was natural human IL-2. The fourth type used was the well-known Aldesleukin, which is produced in *Escherichia coli* (*E. coli*), and thus not glycosylated.

### 2.3. Cell Proliferation Assays (WST-Assay)

For cell proliferation assays, water soluble tetrazolium (WST) 1 was used in a standardized protocol [53]. The results were analyzed by a spectrophotometer at wavelengths of 450 and 630 nm.

A quadripartite standard curve as well as a blank triplicate and negative control triplicate were prepared, all of which were prepared with a suitable medium without additional IL-2. The CTLL standard curve medium (RPMI 1640 HEPES, 10% FCS, 1% Pyruvat) was augmented with 500 IU IMS-Research per well. All the tissue cell samples were prepared as technical and biological triplicates for urothelial cells, fibroblasts, and its co-culture in a cell concentration of 5 × 103. All four IL-2 types were used in four concentrations (100, 250, 500, 1000 IU/mL). The concentrations used in the protocol cover the typical range used in cell culture (100 IU, 250 IU, 500 IU, 1000 IU). They are per ml and are standard concentrations used to activate immune cells in cell culture [54].

While co-culture and urothelial cell assay were prepared with REM, fibroblast tests were performed with DMEM 10% FCS. The functionality of each IL-2 type was confirmed by positive controls with CTLL proliferation assays using 500 IU per well of each solution and a cell number of 1 × 10^3^ and 5 × 10^3^ cells. Co-culture samples were prepared using half of the cell amount of each type in a ratio of 1:1. On day 0 plates for day 0, 3, and 6 were prepared and cultured. Standard curves were prepared freshly every time they were measured.

### 2.4. Apoptosis and Necrosis Analysis

Cell batches were cultured as mentioned above up to second passage and then detached with Accutase^®^. Afterwards, cells were seeded onto coverslips in six-well-plates in a concentration of 5 × 10^3^ for day 3 and 6 analysis and 5 × 10^4^ for day 0 measurement. Every time immunofluorescence was analyzed, new slips were prepared. Tests were performed in technical and biological triplicates for the single culture of urothelial cells and fibroblasts, respectively. IMS-Research and Aldesleukin were used in a concentration of 500 IU/mL. Every time they were measured (day 0, 3, 6), a negative control was prepared.

The staining protocol was performed with the “Healthy cells assay” kit by Promokine according to the manufacturer [55]. The detection of a red nucleus combined with a green outer cell membrane was defined as apoptotic processes, while the detection of only a red nucleus was defined as necrosis. To prevent massive fluorescent overload, a halved concentration of Ethidium Homodimer III was used. Immunofluorescent imaging and visual counting were conducted immediately afterwards in a magnification of 60× with the Zeis Axioscope A1-KMAT.

### 2.5. CD25 Analysis

The detection of CD25 was performed by immunofluorescent staining. Tissue cells were extracted and cultured as mentioned above. Porcine T-lymphocytes were cultured in a culture flask and 20 mL RPMI 10% FCS + 500 IU/mL IMS-Research were added. All samples were created as technical and biological triplicates.

All three batches of fibroblasts, urothelial cells, and T cells were subsequently seeded onto coverslips in six-well plates and cultured with an IL-2 infused medium for two to four days. Two hours before fixation, samples were stimulated with a new IL-2 infused medium. Afterwards, they were fixed with 4 mL ice-cold methanol 96% for 15 min at −20 °C.

Immunofluorescent staining was performed following the same immunofluorescent staining protocol as mentioned above using anti-porcine CD25 of mice as primary antibodies and anti-mouse-antibodies linked to Cy3 (Alexafluor 488) as secondary antibodies. Immunofluorescent imaging and visual counting were conducted immediately afterwards.

## 3. Results

### 3.1. Cell Proliferation Assays (WST-Assay)

**Figure 1:**
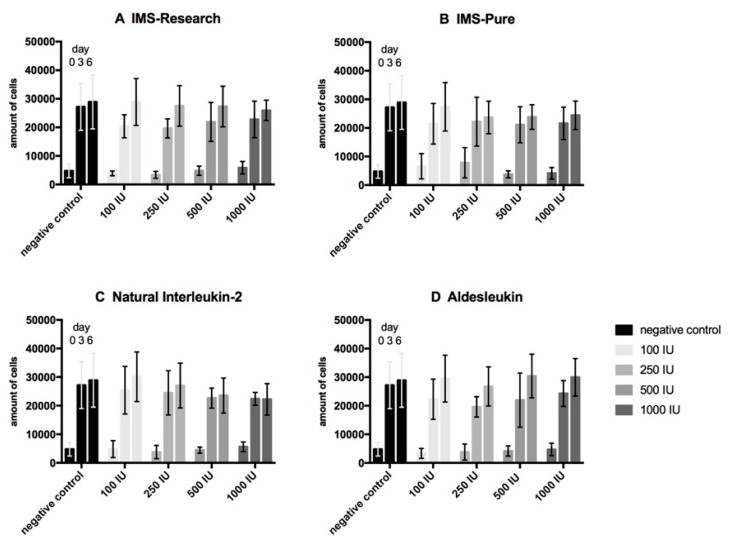
Proliferation assay of urothelial cells. Mean values of technical and biological triplicates were analyzed. Cells were tested with four different interleukin-2 (IL-2) types (IMS-Research, (**A**); IMS-Pure, (**B**); Natural IL-2, (**C**); Aldesleukin, (**D**)) in four different concentrations (100 IU/mL, 250 IU/mL, 500 IU/mL, 1000 IU/mL), and compared to a negative control. The absolute number of cells on day 0, 3, and 6 is displayed in each group of bars of the same color. IU, international units.

**Figure 2:**
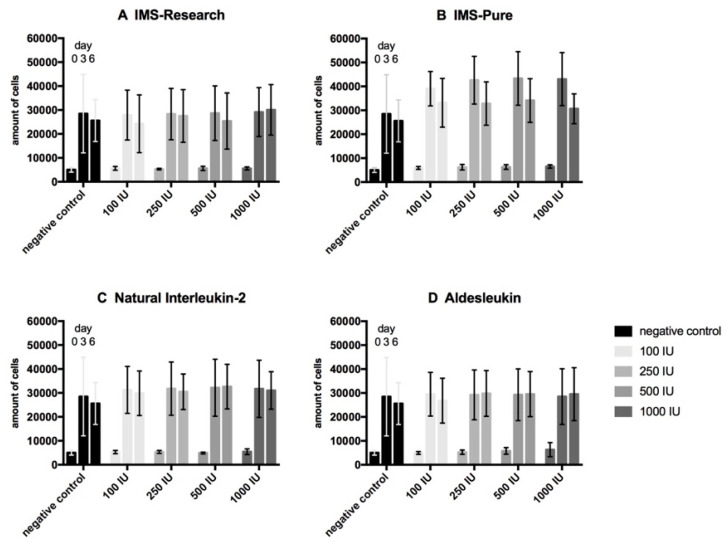
Proliferation assay of fibroblasts. Mean values of technical and biological triplicates were analyzed. Cells were tested with four different IL-2 types (IMS-Research, (**A**); IMS-Pure, (**B**); Natural IL-2, (**C**); Aldesleukin, (**D**)) in four different concentrations (100 IU/mL, 250 IU/mL, 500 IU/mL, 1000 IU/mL), and compared to a negative control. The absolute number of cells on day 0, 3, and 6 is displayed in each group bars of the same color.

**Figure 3:**
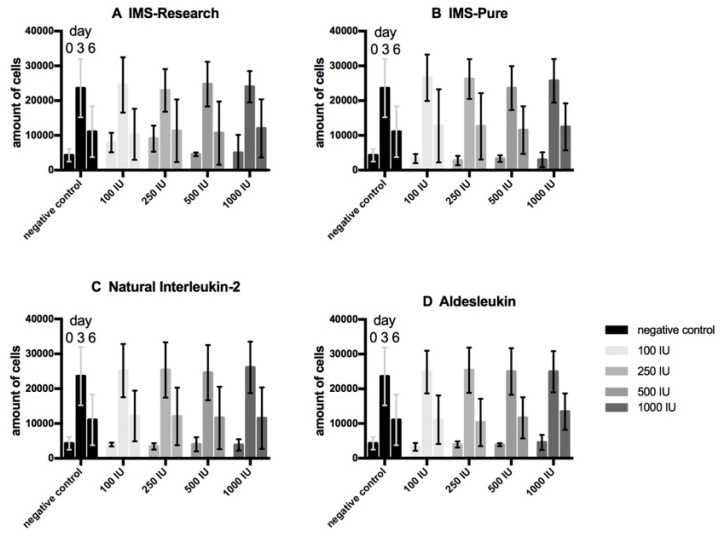
Proliferation assay of co-cultures. Mean values of technical and biological triplicates were analyzed. Cells were tested with four different IL-2 types (IMS-Research, (**A**); IMS-Pure, (**B**); Natural IL-2, (**C**); Aldesleukin, (**D**)) in four different concentrations (100 IU/mL, 250 IU/mL, 500 IU/mL, 1000 IU/mL), and compared to a negative control. The absolute number of cells on day 0, 3, and 6 is displayed in each group of bars of the same color.

Proliferation assays of urothelial cells (Figure 1) present a comparable development for our immunotherapy candidate IMS-Research as well as the other three tested types of IL-2 containing substances (IMS-Pure, natural IL-2, and Aldesleukin) and the negative control regarding the progression of the cell amount. All the samples display a threefold expanse in the first three days and just a small multiplication to day 6 of testing. Under the incubation with IL- 2, no significant deviation from the negative control can be detected. Proliferation assays of fibroblasts (Figure 2) present a similar development in the first half of testing by more than quadrupling cell concentration. Only IMS-Pure influenced cell number values are much higher than those of the other three IL-2 types and present an increasing amount correlating with increasing IL-2 concentration. IMS-Pure, which had the largest expanse to day 3, registered the biggest loss of cells up to day 6. All in all, only for IMS-Pure is a slight difference from the negative control detectable, although it is not significant. All the other IL-2 types display no deviation from the negative control. Proliferation assays with co-cultures of urothelial cells and fibroblasts (Figure 3) overall present the smallest amount of cell expansion during the testing period. Each sample consistently shows a similar development of cell concentration compared with the negative control. Furthermore, all the samples including the negative control display a drastic drop of cell number from day 3 to 6 and all in a high variance of standard deviation. Altogether, samples of IL-2 types do not deviate from the negative control regarding cell population development.

### 3.2. Detection of Apoptotic and Necrotic Processes

**Figure 4:**
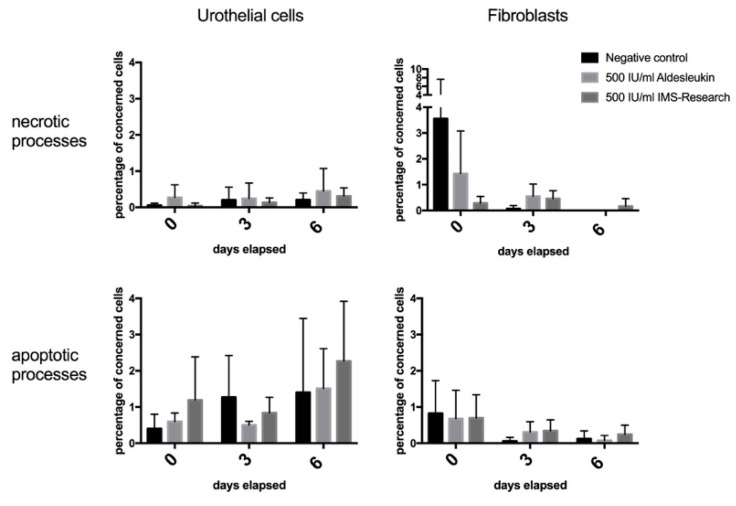
Apoptosis/necrosis of urothelial cells and fibroblasts. The assay was conducted and data were evaluated and displayed in percentage of concerned cells (Y-axis). The development of this percentage was displayed over a test period of six days (X-axis). Aldesleukin and IMS-Research in a concentration of 500 IU/mL were used in comparison with the negative control. Fibroblasts and urothelial cells in single culture were tested and displayed.

**Figure 5:**
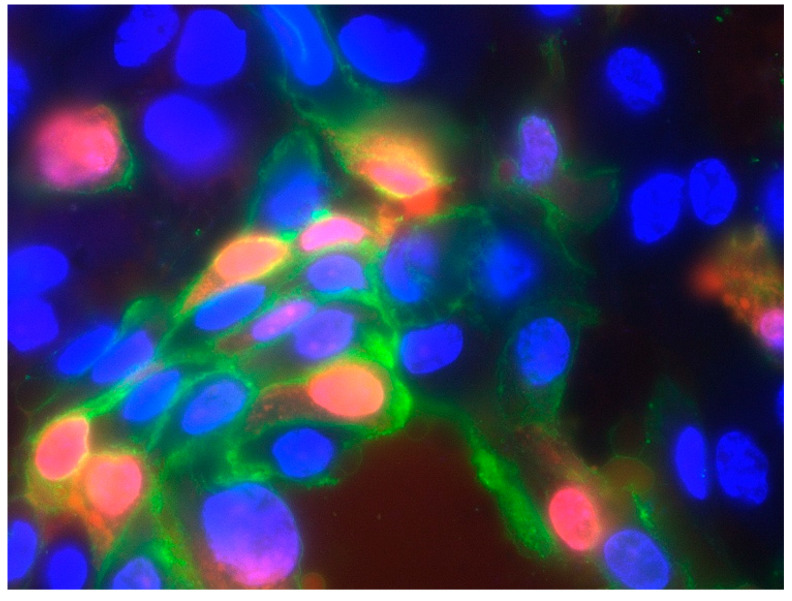
Detected apoptotic urothelial cells on day 6, showing a red nucleus and the green Annexin V detection of the outer membrane specific apoptotic processes. Imaging was performed in a magnification of 60× with the Zeis Axioscope A1-KMAT.

**Figure 6:**
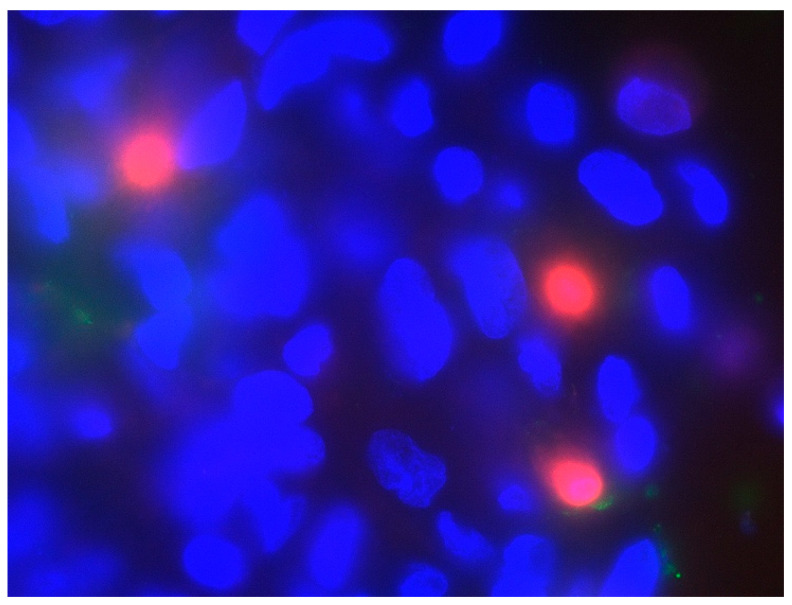
Detected necrotic fibroblasts on day 0, presenting necrotic cell death processes. Imaging was performed in a magnification of 60× with the Zeis Axioscope A1-KMAT.

We generated data for the development of cell death processes under IL-2, displayed in Figure 4, by performing the immunofluorescent staining of specific markers for apoptosis (Figure 5) and necrosis (Figure 6). Our analysis shows that red stained cell nucleus, implicating necrosis, are rarely identified in urothelial cell culture, with no difference between IL-2 types and the negative control. Their number rises slightly to day 6, but stays under 1%. Fibroblast culture, especially the negative control, presents the highest values for necrotic cells on day 0 (about 3.7%) and falls to almost 0% on day 6. Apoptotic processes, detected via the green fluorescent antibody against Annexin V of the outer membrane (Figure 5), are more likely presented. In particular, urothelial cells present an increasing tendency in all three specimens, with the highest values for IMS-Research and lowest for negative control, except for day 3. Fibroblasts, on the other hand, display an overall falling tendency of apoptotic cells in all three specimens from day 0 to day 6, invariably remaining below 1% in total.

### 3.3. Detection of the Alpha Subunit of the IL-2 Receptor (CD 25)

**Figure 7:**
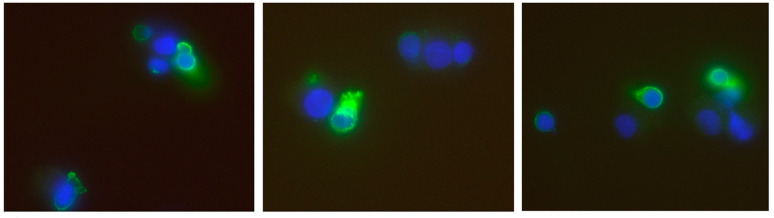
Images of the positive control with stained porcine T-lymphocytes with a primary antibody against CD25 and a secondary antibody crosslinked with a fluorochrom (Alexa Fluor 488), detected with immunofluorescent microscopy (60×).

CD25 was identified by staining cells grown on slides with a specific immunofluorescent anti-porcine CD25-antibody. All tested tissue cells, either fibroblasts or urothelial cells, out of all three cell lines, were 100% negative for this antibody. In contrast, the positive control with T lymphocytes partially stained positive for their activation marker (Figure 7). Under IMS-Research influence, about 11.5% of T cells stained positive, 11.25% of T cells cultured with IMS-Pure, 11.94% of T cells cultured with natural IL-2, and only 6.6% under the influence of Aldesleukin.

## 4. Discussion

“Maximal efficacy by minimal harm” is the superior goal of cancer treatment. The effectiveness of IL-2 application for urothelial cell carcinoma, that is, NMIBC, has been proven on various occasions [41,42,43,44,45,46]. Nevertheless, many occurring adverse events support the hypothesis that not only cancer cells are affected by IL-2 impact [20]. The influence of IL-2 on healthy urothelial cells and its surrounding stroma, in particular, has not been analyzed yet. This leads to an insufficient assessment of the side-effect profile of IL-2. The aim of this study is to solve this problem and diminish this gap of missing information about its direct impact on bladder tissue during intravesical application, thereby contributing to its implementation as a safe alternative in NMIBC therapy.

Earlier investigations not only found IL-2 application to be effective in treating bladder cancer, but also showed the expression of the interleukin-2 receptor (IL-2R) on many different tissue cell lines of the body [12,13,14,15,16,17,18]. Most of them were types of fibroblasts, thus having their origins in the mesenchymal cell line. However, Gerritsma et al. found renal epithelial cells to express an IL-2R [12]. The latest investigations have also detected functional heterotrimeric IL-2R on vascular smooth muscle cells [56]. On the basis of these findings, we analyzed, in in vitro experiments, the proliferation of porcine urothelial cells and fibroblasts in single and co-culture under IL-2 influence, the effect of IL-2 on cell death processes, and the potential expression of an IL-2R.

For this purpose, four different types of substances containing IL-2 were used for our analysis. First of all, we analyzed our new IL-2 immunotherapy candidate called IMS-Research and its highly purified version, IMS-pure. IMS-Research as well as IMS-Pure contain human recombinant IL-2 produced in Chinese hamster ovarian (CHO) cells. They provide a quantity of glycosylated as well as non-glycosylated IL-2 isoforms, including a glycosylated tetrasaccharide, a glycosylated trisaccharide, and a non-glycosylated isoform. This pattern of glycosylation of recombinant synthesized IL-2 is most likely comparable to physiologically existing human IL-2 and is also very stable [57]. Additionally, Aldesleukin (or Proleukin) is a recombinant IL-2 variant produced in *Escherichia coli* (*E.coli*) strains [34] and the only type internationally approved and used in common therapy in clinical practice [34]. Aldesleukin, a desalanyl-1, serine-125 human interleukin-2, has been produced by genetic engineering techniques used to modify the human IL-2 gene, and it differs from native interleukin-2 in the following ways: (a) Proleukin is not glycosylated because it is derived from *E. coli*; (b) the molecule has no N-terminal alanine; (c) the molecule has serine substituted for cysteine at amino acid position 125; and (d) the aggregation state of Proleukin is likely to be different from that of native interleukin-2. The fourth substance used was natural IL-2 (nIL-2) produced by mitogen-activated lymphocytes, which were isolated from healthy human blood donations. This last type of IL-2 presents the greatest similarity of all the IL-2 types used to natural human IL-2, providing a purity level of 99%, and is stabilized by human serum albumin and a sodium-phosphate buffer.

Contrary to our expectations, on the basis of the literature, the results arising from the WST-proliferation assays for urothelial cells, fibroblasts, and its co-culture showed no deviation of cells cultured in IL-2 containing medium compared with the negative control in either of the concentrations used. Mostly, growth curves displayed an asymptotic course. Urothelial cells presented a strong increase to day 3, but only a slow development in the second half until day 6. Fibroblasts also showed a large expanse in the first half followed by a tendency to stagnate or slightly decrease in the second half. Co-culture tests had risen to day 3 and fallen sharply to day 6 in the number of cells. While differences were noticeable between urothelial cells and fibroblasts, as well as their co-culture, within one cell line, development of proliferation was similar to the negative control cultured without IL-2 in the cell culture medium. As our positive controls with IL-2 dependent cytotoxic T lymphocytes showed enhanced proliferation by adding IL-2 infused medium of all types, the activity of our IL-2 solutions can be said to be proven. These findings lead to the suggestion that the sole IL-2 effect does not have a negative impact on cell vitality of the in vitro cell cultures of urothelial cells, fibroblasts, and its co-culture.

The noticeable absence of any impact of IL-2 may have been caused by a short half-life of the cytokine. In the literature, the in vivo half-life of IL-2, relevant for physiological processes as well as therapeutical approaches, was examined and revealed a bi-exponential course with peaks at about 13 and 85 min in serum [25]. Our test settings did not refer completely to a constant intravesical installation of IL-2, but rather episodic. Further investigations will have to clarify if there is a diverging impact when continuously applied and if anti-IL-2 antibodies or the in vivo immunologic microenvironment of the human bladder might prolong the half-life, as recent studies suggest [58].

Furthermore, the sigmoidal growth curve mentioned is associated with the proven behavior of cells during cell cycle, also including a death phase or quiescence due to contact inhibition [59,60,61]. As the literature also displays pro-proliferative effects of the interaction between urothelial cells and fibroblasts [62,63,64], and our co-culture results present the most drastic decrease of all samples, growth limitation due to lack of nutrition, space, and cell contact inhibition can be presumed as the cause for the growth curve rather than IL-2 impact, especially as there is no difference from negative controls.

In order to confirm whether our results referred to physiological apoptotic processes due to lack of nutrition or space or necrotic processes of toxic effects of IL-2, we implemented a staining protocol for apoptotic and necrotic processes. As a result, apoptotic processes were immunohistochemically detected via fluorescein linked Annexin V on the outer membrane [65]. The staining results displayed diverging results for fibroblasts and urothelial cells. While urothelial cells present increasing percentages of apoptotic cells over the testing period, more affected under IMS-Pure, fibroblasts display a higher impact of necrotic procedures, especially in negative control on day 0. Both cell lines display slightly more necrosis impacted cells when cultured with Aldesleukin compared with IMS-Pure. Interestingly, with regard to proliferation assays, fibroblasts are shown to be more concerned by stagnation or even a decrease in cell numbers, while apoptosis/necrosis tests do not correlate with these findings and rather display a negligible amount of cell death concerned cells, especially on day 3 and 6. Regarding the highest value of necrosis on day 0 for fibroblasts, an inaccurate treatment during IL-2 application or staining has to be taken into consideration. Further studies with preferably more objective analysis procedures and higher sample sizes have to be performed. The decrease especially in urothelial cells might also be related to physiological developments of growth curves. Zwietering et al. stated that the death phase was one part of a physiological growth curve [59], following a proliferation phase. Subsequently, death and proliferation fluctuate constantly and form an overall plateau state. Next to this cell cycle rationale, the entrance into the reversible quiescence by contact inhibition [60,61] or into the irreversible senescence state by oxidative stress or replication failure [61,66,67] is conceivable as one outcome of our results. Concluding that, especially for urothelial cells, the fall in cell numbers was more a result of processes without any IL-2 influence, it might be a physiological process of cell contact inhibition by reaching confluence and restricted free area. This thesis would concur with the fact that samples with lower third day values did not display decreases as drastic as those with extremely high third day values. The literature shows that this phenomenon is recognized on a widespread basis [61,68]. Puliafito et al. even declares a complete stop of mitotic processes caused by contact inhibition and following interactions of cells [61].

All in all, these results confirmed our hypothesis of there not being a detectable toxic effect of IL-2, especially during the course of the testing period. These findings also correlate with recent literature. While systemically applied IL-2 was proven to lead to severe side effects like the vascular leak syndrome, diarrhea, or oliguria [26,69], clinical trials using intravesical or other local application forms of IL-2 presented minimal adverse events [20,45,46,70,71,72,73].

In order to fully prove the absence of any IL-2 impact on tested cell lines, the expression of an IL-2R was detected. The most significant limitation was the absence of a suitable anti-porcine antibody against both other subunits CD122 or CD132. The only retrievable agent was an anti-porcine antibody against CD25. Even though not all the tissues analyzed for IL-2R revealed the demonstration of an alpha chain, different tissue sources like human fibroblasts, primordial germ cells, and human renal proximal tubular epithelial cells presented positive staining for CD25 in the studied literature [12,13,18].

Although research implies the potential of expressing the IL-2R, neither our proliferation assays nor our receptor subunit detection displayed the presence of the alpha subunit. Hence, the absence of the high affinity tricomplex IL-2R on porcine transitional epithelium as well as porcine bladder fibroblasts can be suggested. Nevertheless, the absence of CD25 does not confirm the non-existence of any IL-2R. In particular, the alpha chain tested was stated to be partially absent when inactivated [74]. Scientists found that the high affinity receptor formation in T cells requires a co-stimulation via a T cell receptor [75] or other cofactors to contribute to proliferative pathways [12,17,75,76,77], assuming that the lack of the co-stimulation of factors or receptors might have led to failed expressions. On the other hand, the latest results lead us to presume that the absence of CD25 might be related to a better anti-tumor outcome and less adverse effects occurring. Krieg et al. [78] claimed adverse effects as being referred to CD25 binding on endothelial cells. This is supported by Carmenate et al. [79], who found a reduced binding to CD25 to be beneficial for anti-tumor effects. Levin et al. [80] found CD122 stimulation to enhance the proliferation rate of T and NK cells and thus improve anti-tumor effects. Thus, further investigations for the existence of CD122 in particular and CD132 on urothelial cells of porcine and particularly human origin have to be performed with suitable antibodies.

The immunologic network is highly complex with many pathways leading to opposite effects, despite sharing the same molecules, depending on the apparent conditions. For example, Yan et al. found cell circle networks to share many molecules like STAT proteins or NFKB, thus they were described to have pro- and anti-apoptotic capabilities [81]. This interfering molecule network is presumably a big part of the reason that cytokines like IL-2 are able to switch in outcome. Gonzales-Garcia and colleagues have agreed with this hypothesis after investigating the different signaling of the beta and gamma chains and state that apoptosis and proliferation share one pathway system [82,83]. For this reason, further investigations including in vivo immunological networking processes need to be implemented in order to fully analyze the impact of IL-2 during its usage in vivo.

As far as BCG-therapy associated limitations are concerned, the importance of IL-2 as a candidate for cancer immunotherapy is becoming even more significant. Primarily, a large proportion of patients (roughly 75%) under BCG-therapy can be claimed to be nonresponsive as they experience a recurrence within 5 years after adequate therapy [84]. Moreover, the rate of reported side effects of up to 70% is extremely high [85,86,87,88], thus affecting the quality of life for patients. In addition, the duplication of BCG currently seems to be limited as a result of grievances in production firms and an increased demand. Differences in the molecular structure of BCG-strains worldwide based on a long history of sub-cultivation and the ongoing changes in the genetics of all these substrates might lead to a variation in the efficiency of the therapy in the long term [89].

The anti-tumor mechanism of a BCG therapy works by promoting the immune system of the host. Antigen-presenting cells produce cytokines and chemokines, which attract different cells of the immune system, thus supporting the immunological anti-tumor microenvironment of the bladder [90]. In particular, the production of IL-2 was said to be associated with a significantly improved response to BCG therapy [91]. IL-2 recruits cytotoxic T cells and promotes converting naive to effector T cells, thus supporting the immune response by “targeting a defective p53 pathway” [92,93]. Investigations used these approaches and found IL-2 to enhance the effectiveness of low-dose BCG therapy [94], considering IL-2 to also be effective in a combined treatment. In addition, Doersch et al. stated in 2017 that the influence of IL-2 is decisive for healing wounds [7], which is a critical part of the therapy.

## 5. Conclusions

Many studies have confirmed the effectiveness of IL-2 applied intravesically for bladder cancer immunotherapy. Additionally, minimal to no side effects at all were reported when applied locally. Our results indicate the harmlessness of the IL-2 immunotherapy candidate used for porcine urothelial cells and fibroblasts of the bladder in in vitro experiments. Therefore, our results are one more step towards a fully covered understanding of the impact of IL-2 on tissue cells regarding the implementation as an immunotherapy. As the use of porcine tissue and human IL-2 provides restricted validity for in vivo application, further investigations concerning the usage of healthy and pathological human cell lines of the bladder have to be established, particularly focusing on the impact of the non-reproduceable in vivo microenvironment and networks.

## Abbreviations

BCG	Bacillus Calmette-Guerin
CHO	Chinese hamster ovarian
CO_2_	Carbon dioxide
CTLL	Cytotoxic T lymphocyte
DMEM	Dulbeccos modified Eagles medium
E.coliFCS	*Escherichia coli*
IFN	Fetal calf serum
IL-2	Interferon
	Interleukin-2
IL-2R	Interleukin-2 receptor
IU	International units
MAPK	Mitogen-activated protein kinase
NMIBC	Non muscle-invasive bladder cancer
Pi3K	Phosphatidylinositol-3-kinase
P/S	Penicillin/streptomycin
PBS	Phosphate buffered saline
REM	Renal epithelial medium
RPMI	Roswell park memorial institute
SmPC	Summary of Product Characteristics
TNFa	Tumor necrosis factor alpha
WST	Water soluble tetrazolium
